# The unseen toll: excess mortality during covid-19 lockdowns

**DOI:** 10.1038/s41598-023-45934-2

**Published:** 2023-10-31

**Authors:** Florian Ege, Giovanni Mellace, Seetha Menon

**Affiliations:** 1 Interdisciplinary Centre on Population Dynamics, University of Southern Denmark, Odense, Denmark; 2 Department of Economics, University of Southern Denmark, Odense, Denmark

**Keywords:** Public health, Infectious diseases

## Abstract

In March 2020, in an attempt to slow the spread of Covid-19, several countries intervened by imposing strict lockdown measures that limited contact among people. In contrast, Sweden decided to not implement a mandatory lockdown and instead allowed people free choice on whether or not to follow the government recommendation to limit contact with others. Using the Synthetic Control Method, we estimate the causal effect of not implementing a mandatory lockdown in Sweden in the period from the end of February 2020 to the end of September 2020, a time when vaccines were as yet not available. We find that not imposing a mandatory lockdown resulted in a lower reduction of mobility and a substantial increase in mortality. Our results indicates that up to about 4411 of the 46554 deaths registered in Sweden during this period could have been avoided had Sweden implemented a mandatory lockdown. These results remain consistent when using two additional state-of-the-art estimation methods; the augmented synthetic control method and synthetic difference-in-difference.

Since the wake of the Covid-19 pandemic, a phenomenal amount of research has been undertaken on the effectiveness of various non-pharmaceutical interventions (NPIs) to reduce infection rates, hospitalizations and deaths. However, the existing evidence is contradictory, and the methodology employed in several scientific papers have subsequently come into question^1–3^. Estimating the effect of NPI’s is particularly challenging, for two reasons. First, we do not know to what extent people would have reduced their contacts in the absence of NPIs. Second, the levels of enforcement, compliance, and a host of relevant factors such as population densities, viral prevalence, diffusion dynamics, hospital beds available, testing frequency and/or recording of infections and deaths, can be very different from one region to the other. Unsurprisingly, the estimated effects of NPIs also vary greatly in the existing literature, based on the context studied, the statistical methodology used, and the Covid-19 outcome in question^4,5^.

Thus, the debate on the effectiveness of NPIs in fighting the pandemic is far from resolved^6^. The UK, is currently undergoing a public inquiry over the government’s handling of covid, with experts divided as to whether a lockdown could have been avoided, versus those who believe lives were lost due to a delayed lockdown^7^. Since humanity can expect pandemics to occur more frequently in the future than they have done in the past^8^, and because we are better positioned to leverage more and better quality data, we contribute to this debate by estimating the causal impact of not implementing a lockdown in Sweden on excess death outcomes using data from the Human Mortality Database (HMD) and synthetic control methods (SCM). Compared to the average number of deaths in the previous 5 years, 7144 more Swedes died in the year 2020, this is a substantial number in excess in a country of approximately 10.3 million inhabitants. While an increase in excess deaths has been a common phenomenon around the globe during the pandemic, a quick glance at the numbers suggests that Sweden experienced substantially more excess deaths when compared to similar countries.

Sweden posed very similar policy recommendations as most other countries, yet fell short of enacting them as mandatory, instead choosing to encourage voluntary compliance by citizens. This offers an interesting case study for comparing the effectiveness of compulsory versus voluntary NPIs. For this reason, three studies on Covid-19 that use SCM have focused on Sweden as the treated unit. Using a donor pool of European countries as a counterfactual for Sweden, Born et al. find that infections and deaths directly attributable to Covid-19 in Sweden, would have been reduced by one half and one third, respectively, had Sweden imposed a mandatory lockdown^9^. They also find that the effect of the lockdown materialises with a delay of 3–4 weeks. Cho^10^ finds that infection cases would have been reduced by almost 75% and excess mortality would have been reduced by 25 percentage points had Sweden imposed a lockdown. The author also finds that the impact of NPIs become visible after a time lag of approximately 5 weeks. In contrast to these two studies, a more recent SCM analysis, finds that a lockdown in Sweden would have had sizable effects within 1 week^11^, and that the longer delay estimated by the two previous studies is driven by the extremely low testing frequency that prevailed in Sweden in the early months of the pandemic. The authors emphasise the need for examining multiple indicators of Covid-19 such as daily Covid-19 deaths corrected to account for the downward bias in the measurement of such deaths due to miss classification and the test positivity rate.

We extend this literature in four ways. First, the focal point of our analysis is excess mortality and not number of infections, as in Born et al.^9^ and Cho^10^. Our study also differs from Latour et al.^11^ who use a variety of indicators but fall short of using weekly excess mortality as an outcome. Data on Covid-19 infections has several shortcomings such as measurement error, and crucial to this research, detected cases are not independent of the number of tests. By using excess mortality as an outcome we are able to leverage a longer time horizon in the pre-intervention period, which ensures that excess mortality dynamics before the imposition of mandatory NPIs is comparable between Sweden and our counterfactual, synthetic Sweden. Second, we show that our results are consistent by implementing two additional state of the art methods: the augmented synthetic control^12^ and the synthetic difference-in-differences^13^. The former allows for negative weights with the advantage of potentially improving pre-treatment fit at the cost of a higher risk of over-fitting, while the latter combines the advantages of SCM and difference-in-differences weighting not only control units but also pre-treatment periods. Third, in contrast to prior work which only examines the effect up until the end of June 2020^10^ or August 2020^9^, we extend the time frame of analysis to roughly the end of September, 2020. Fourth, as previous work has shown that different models for computing excess deaths produce very similar weekly patterns but may disagree substantially on the level of excess deaths^14^, we ensure our results are not sensitive to the model used to predict excess deaths.

## Results

We use the Human Mortality Databases’ Short Term Mortality Fluctuations (STMF)^15^ data (See “Placebo tests” for details) and synthetic control methods with Sweden as the treated unit to estimate the causal effect of not implementing a lockdown on excess deaths. We examine two outcomes to measure excess deaths. We use (accumulated) weekly excess mortality which are based on the 5 year average death count model, i.e., starting from the first week of November 2019 we compute the difference between weekly deaths and the average number deaths in the previous 5 years in the same week, accumulated at each subsequent week.

Recent work on the viability of different model specifications for estimating excess deaths^14^ finds that, out of 9 different estimation methods considered, a 5 year average death count model has the strongest upward bias of excess, while a 5 year average death rate model has the strongest downward bias of excess. Accordingly, it is suggested that studies using excess death as an outcome ought to test for effects using estimates of excess deaths from both these models. If effects exist using the outcomes derived by both models, they are likely to be robust against bias introduced due to the excess death estimation approach chosen. While the death rate model adjusts for increasing deaths due to population aging, both models do not adjust for decreasing deaths due to mortality improvements^14^. Previous work has shown that when accounting for improved life expectancy, post-pandemic excess mortality in Sweden is higher^16^, thus our findings are likely to be a conservative estimate of the effect of not locking down.

The idea behind SCM is to construct a counterfactual version of Sweden (synthetic Sweden) as a weighted average of the outcome of the control units (in our case all the other countries in the STMF data for which we observe deaths and death rates from November 2014 to the end of 2020). The weights are chosen such that the distance between the observed characteristic of the treated unit and the control unit is minimized in the pre-treatment period. If the chosen weights make the pre-treatment trends of the outcome of the treated and the synthetic control overlap, under the assumption that this overlap would have continued in the treatment period in the absence of the treatment, we can use the synthetic control to estimate what would have happened to the treated unit had not it received the treatment. By using SCM, we ensure that our synthetic control unit (Synthetic Sweden) evolved in a comparable way to our treatment unit (Sweden) before the implementation of mandatory lockdown measures and therefore we can control for all confounders (including time varying confounders) which depend on pre-treatment differences. As a robustness check, we also estimate the effect of not implementing a strict lockdown in Sweden by the augmented synthetic control method^12^, which allows for negative weights and better pre-treament fit, and by the synthetic difference-in-differences (Synth-DiD)^13^, which construct weights for pre-intervention periods as well as for each unit in the donor pool. The advantage of Synth-DiD is that it allows to account not only time varying pre-treatment confounders as SCM but also for unit specific time invariant confounders as the standard DiD, combining the strengths of the two methods. For each outcome considered, we optimise the weights separately.

In Sweden, the number of infections surpassed a threshold of one infection per one million inhabitants on the 29th of February, 2020. The timing at which countries implemented a mandatory lockdown varies between 13 to 24 days from this threshold. As the treatment is not implementing a mandatory lockdown, a hypothetical treatment start date has to be chosen. For our main specification, we provide a conservative estimate choosing a slow response (4 weeks), and provide an alternative scenario for a rapid response (2 weeks) as a robustness check.

Synthetic Sweden is constructed based using the weeks leading up to the start date which is Week 1 (1st week of November, 2019) to Week 22 (4th week of March, 2020) for our main specification. Our donor pool consist of 29 countries: Austria; Belgium; Bulgaria; Canada; Switzerland; Czechia; Denmark; Germany; Spain; Estonia; Finland; France; Croatia; Hungary; Iceland; Israel; South Korea; Italy; Luxembourg; Lithuania; Latvia; Netherlands; Norway; New Zealand; Poland; Portugal; Russia; Slovenia; and Slovakia.

For our main outcome of weekly excess deaths/100,000, the weights received by each country which contribute (at least 1%) to construct synthetic Sweden are: 12.3% Belgium; 19.6% Denmark; 3.8% Finland; 17.5% Lithuania; 29.6% Norway; and 17% New Zealand (the remaining 0.2% weights are assigned to the other countries in the donor pool). For our secondary outcome of weekly excess rate, the weights received by each country which contribute (at least 1%) to construct synthetic Sweden are: 52.4% Norway; 36.5% Lithuania; 4.8% Latvia; and 6.1% New Zealand (the remaining 0.2% weights are assigned to the other countries in the donor pool). Synthetic Sweden is assumed to have a lockdown lasting approximately 10 weeks, which is the average period for which the 6 donor countries imposed the mandatory lockdown. Section “Leave-one-out results” shows that the results are robust to alternative donor compositions.

**Table 1:** Balance on baseline Characteristics.

	**Sweden**	**Synthetic**	**Donor**	**Variable**
		**Sweden**	**Average**	**Weights**
Deaths (Average)	-6.7	-6.7	-4.1	18.2 %
Deaths (Week 22)	-14.3	-14.3	-7.8	24.3 %
Deaths (Week 21)	-16.4	-16.1	-10.6	17.2 %
Deaths (Week 20)	-17.1	-16.5	-12.1	2.6 %
Deaths (Week 19)	-15.4	-15.8	-12.3	19.2 %
Deaths (Week 18)	-14.6	-14.5	-11.2	13.1 %
GDP per capita	50683	51234	43700	3.7 %
Cardiovascular deaths	138.6	158.8	192.5	1.2 %
Life expectancy	83.0	81.0	80.3	0 %
Population density	24.8	91.6	145.1	0.2 %
Urban population	88.0	84.0	76.1	0.3 %

Table 1 reports descriptive statistics and weight of the predictors used to construct synthetic Sweden. Pre-treatment values of the outcomes contribute the most when constructing the weights, while other control variables contribute less to the construction of synthetic Sweden. Deaths due to cardiovascular disease and population density are lower in Sweden as compared to synthetic Sweden. However these variables also receive low weights and at worst would likely bias our results towards zero. Due to the difficulty to construct credible measures of climate that capture variation at the country level, especially for countries with vast territories such as Sweden, we are not able to credibly control for climate. Using the World Bank data catalog that measure average monthly temperature in each country in the period 1961-1999, we find that on average Sweden is colder than Synthetic Sweden. This indicates that controlling for climate would make our estimates even larger.

**Figure 1:**
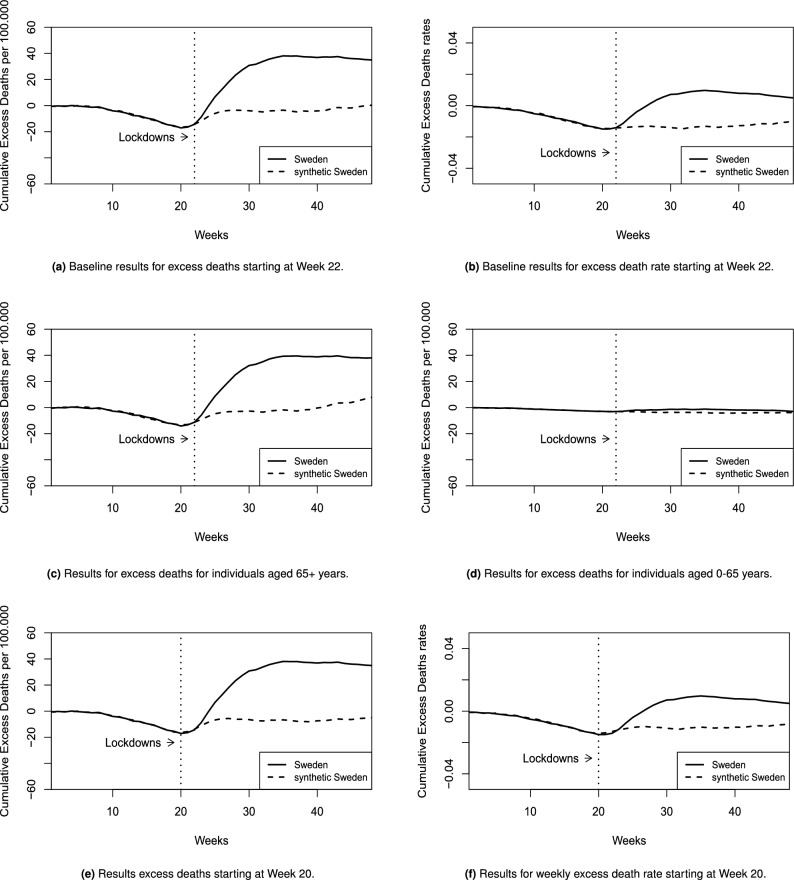
Baseline results, results by age group & results for rapid response.

Panels a and b in Fig. 1 show the results of our preferred conservative estimate of a slow response (4 weeks). We find that the average effect of not implementing a mandatory lockdown in Sweden in our period of analysis, is 33.2 deaths/100,000 inhabitants. This translates to approximately 3420 deaths in Sweden that may have been avoided, had Sweden imposed a mandatory lockdown by Week 22, ie. towards the end of March, 2020. While Sweden and synthetic Sweden start to diverge immediately, we find that the divergence peaks towards the end of June (Week 35) with a size of 42.82 deaths/100,000 inhabitants which translates to approximately 4411 deaths. Further, we compute the SCM estimates separately by sex and find peak effect sizes of 16.68 deaths/100,000 inhabitants for females and 22.49 deaths/100,000 inhabitants for males which is consistent with the documented sex-differences in Covid-19 deaths.

It is well documented that Covid-19 disproportionately affected the elderly, particularly in the case of Sweden where Covid-19 mortality in long-term care facilities were reportedly much higher than its neighbours, 5 fold higher than in Denmark and 11 fold higher than in Norway and Finland^17^. We explore if our results corroborate this by examining cumulative excess deaths by heterogeneous age groups of inhabitants who are 65+ years of age and inhabitants who are below 65 years of age in panels c and d in Fig. 1. The results suggest that our main results are almost fully driven by excess deaths in the 65+ age group, in support of the evidence that ’dry-tinder’(Referring to a hypothetical stock of individuals who are at risk of dying soon^18^) does not account for the level of excess recorded in Sweden^18^.

## Robustness checks

### Rapid response

Panels e and f in Fig. 1 consider a rapid response (2 weeks), such that the hypothetical treatment starts in Week 20, for cumulative death counts and cumulative death rates respectively. Our results are not sensitive to the response time chosen.

### Pre-treatment fit

**Figure 2:**
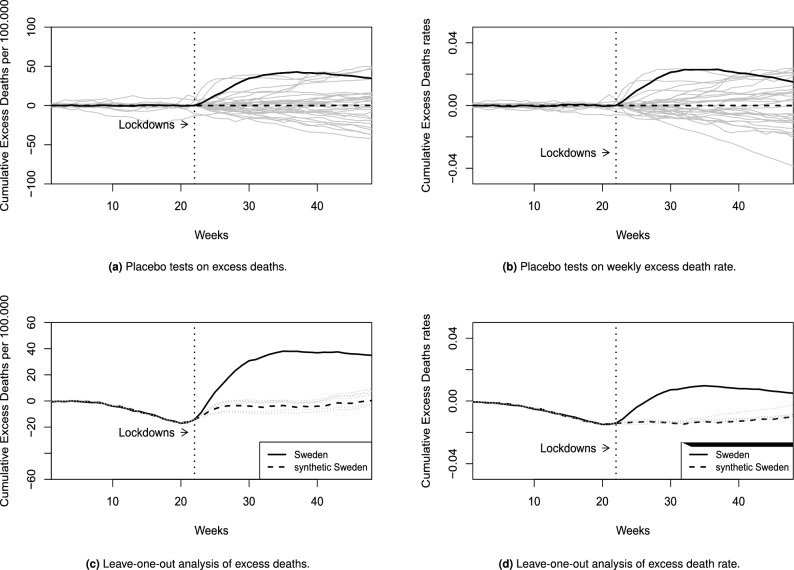
Results for placebo tests and leave-one-out.

The synthetic counterfactual for Sweden closely follows the trend of cumulative excess mortality in Sweden in the weeks leading up to the pandemic as shown in all panels in Figs. 1 and 2. The drop in excess deaths between week 10 and 20 is explained by lower mortality, especially compared to February 2018 which saw significant influenza related excess deaths in Sweden^19^. Extending the pre-treatment period even further by including data from previous years is not possible, as this would have severely limited our donor pool as some countries do not report weekly mortality data in the STMF-dataset for previous years. A further restriction in this regards is that excess mortality is constructed using a 5 year average approach, therefore requiring data not only for the pre-treatment period, but also 5 years prior (2014–2020). Reassuringly, our results are similar when using the augmented SCM, in Sect. “Augmented synthetic control method”, which indicates that the pre-treatment fit in our main specification is good.

### Placebo tests

Panels a and b in Fig. 2 present the results of placebo test in space for the outcomes of excess death counts and excess death rate respectively. The placebo tests are obtained by iteratively applying the SCM to each unit in the donor pool to obtain a distribution of placebo effects. This allows us to infer whether the effect of not implementing a mandatory lockdown in Sweden is extreme when compared to the donor pool’s placebo effects. The figures clearly show that if any of the countries in the donor pool had not implemented a mandatory lockdown, we would seldom find an effect as extreme as that found in Sweden. This shows that our estimated impact of not implementing a mandatory lockdown in Sweden is not likely to be observed by chance, and is clearly driven by the treatment in question.

### Leave-one-out results

Panels c and d in Fig. 2 graphs the two models for the cumulative excess deaths and excess death rate respectively, where step-wise one of the 6 donors is left out. The leave-one-out analysis reveals that the overall findings are independent of individual donor countries with the overall development of cumulative excess deaths in all leave-one-out counterfactual models behaving with rather similar trends. However, they also reveal that for the later part of 2020 (around week 35), marking the onset of what most countries referred to as “the second wave”, the findings do not appear to be clearly independent of individual donors any longer, in particular when examining the excess death rate.

### Augmented synthetic control method

**Figure 3:**
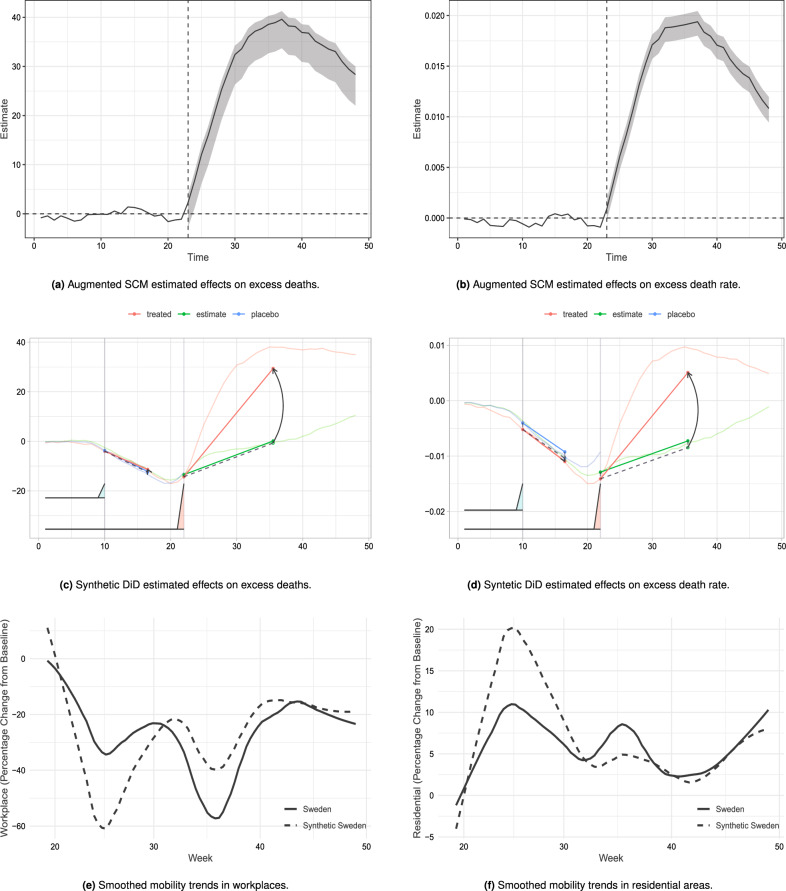
Results for augmented SCM, synthetic difference in difference & mobility trends.

A prerequisite for SCM to be valid is that the pre-treatment outcomes for Sweden closely match the pre-treatment outcomes of synthetic Sweden. As an additional check, we use augmented SCM^12^, which includes a ridge regression predicting post-treatment outcomes among control units, with pre-treatment outcomes and covariates as predictors. Panels a and b in Fig. 3 present the results of augmented SCM on weekly excess deaths and weekly excess death rates respectively. The graphs plot the week-by-week point estimates along with 95% confidence intervals, calculated using the novel jackknife+ method^20^. The confidence intervals are quite small and show a significant positive effect in the entire post-treatment period for both outcomes (see Panels a and b in Fig. 3). The average effect size, for the weeks we examine, is 30.04 deaths/100,000 inhabitants. This is similar to our estimate using the SCM, which indicates that the estimated bias is small and that the pre-treatment fit in our main specification is adequate.

### Synthetic difference-in-difference

We ensure our results are robust to using the very recent advances in the SCM literature, the synthetic difference-in-difference (Synth-DiD) method^21^ which combines the strengths of SCM and difference-in-difference. Synth-DiD introduces weighting for both, cross-sectional units and pre-treatment time periods in the construction of synthetic Sweden, thereby improving on the precision and reliability of the estimator. Similar to SCM, it ensures that pre-treatment trends are matched somehow weakening parallel trend type assumptions typically imposed by DiD methods. Moreover, Synth-DiD, as DiD, allows for the presence of time invariant confounders. Panels c and d in Fig. 3 graph the results of this estimation for the accumulated weekly excess deaths and excess death rates respectively. The shadowed red areas under each pre-treament period represenent the weights that synth-DiD give to each period. For both outcomes the last pre-treatment week receive most of the weight. This is not surprising given that the SCM already achieved a good pre-intervention fit. The difference between the solid red line and the solid green line is the estimated average treatment effect on the treated (ATT) which is 30.06 deaths/100,000 inhabitants with a 95% CI [4.89, 55.24], this confidence interval is obtained using the method described in^21^. The effect is graphically illustrated by the black arrow. The dotted black line represent what would have happened had Sweden imposed a mandatory lockdown under the standard parallel trend assumption adjusting for pre-treatment trends by the synthetic control weighting. The Synth-DiD estimator approximates that 3096 excess deaths could have been avoided, had Sweden implemented a mandatory lockdown. Figure 3 also includes a placebo test which estimate the of a fake intervention that would have occurred at week 10, placebo synth-DiD Sweden is represented by the blue line. The treatment effect of the placebo intervention is very close to zero for both outcomes as shown in both panel c and d.

## Discussion

### What is the magnitude of the effect of not implementing a mandatory lockdown?

With regards to the magnitude of the effect of not implementing a mandatory lockdown, previous studies have found that implementing a lockdown in Sweden could have resulted in a 34% (by May 17th^9^) to upto 47% (by June 30th^11^) reduction in Covid-19 specific deaths. Our estimates are not directly comparable to these as we use measures of excess deaths as outcomes, as opposed to Covid-19 specific deaths. We find a conservative effect size of approximately 30 deaths/100,000 inhabitants in Sweden as compared to synthetic Sweden. This result is slightly higher than^10^, who finds that upto 25% of excess deaths could have been avoided by the week ending June 13th. Our findings also corroborate that of^10^ in that the divergence peaks towards the end of June. The magnitude of this effect remains fairly robust irrespective of the estimation method used.

### Is there a delay in the effect of the lockdown?

We find that Sweden and synthetic Sweden begin to diverge from end of March (Week 22) onward. This is in contrast to early studies^9,10^ that find a 3–5 week delay in the effect of the lockdown on Covid-19 infections which could be an artifact of idiosyncratic testing rates, specifically the extremely slow testing rate in Sweden during the initial phase of the pandemic. Our results are comparable to more recent studies using Covid-19 deaths as outcomes^11^, where effects are seen within a week after the introduction of lockdown in synthetic Sweden.

### Did Swedes reduce their mobility despite the lack of a mandatory lockdown?

We use Google mobility data to compare actual behavioural adjustments between Sweden and synthetic Sweden. Figure 3 presents the Google mobility trends for both, places of work (e) and residential areas (f) for Sweden (solid line) and for our previously constructed counterfactual synthetic Sweden (dotted line). Previous studies suggest a correlation between Covid-19 restrictions and mobility^22^. In comparison to the baseline (first 5 weeks of 2020), it becomes clear that during the first wave of the pandemic (March–June) Swedes spent substantially less time at their workplace (e) and instead spent more time in their residences (f). However, during this first period these changes are substantially smaller than what is observed for the earlier constructed synthetic Sweden. These results are consistent with previous work that show that Sweden only reduced its mobility by approximately half of that of its Nordic neighbours^23^, indicating a more limited behavioural adjustment due to the lack of regulations being enforced as mandatory. During the later half of 2020 reduced adherence and eased restrictions lead to a normalisation of mobility patterns in many countries^24^ with Sweden being an apparent exception, first drastically reducing workplace mobility around week 36. While mobility data is a direct measure of a certain type of individual behavioural adjustments, it is not the only one that might be relevant. Other studies^25^ have highlighted the potential effect of related behaviour adjustments, such as physical separation at the workplace—however, these more detailed individual behavioural adjustments and their potential impact on infection dynamics are outside the scope of this study.

### Can differences in healthcare capacities explain observed excess mortality?

In a 2018 study measuring healthcare access and quality performance, Sweden, unsurprisingly, scored among the leading countries in the World^26^ with very few cases of amenable mortality–deaths from causes that should be avoidable. A later comparison of healthcare capacity^27^ during the early COVID-19 pandemic found that, among the countries studied, Sweden had the lowest capacity of ICU and acute care beds going into the pandemic. The study also finds that Sweden had been able to more than double its capacity during the pandemic. To ensure our results are not driven by differences in healthcare capacity, we included two indicators of healthcare capacity (physicians and hospital beds) as controls in our analysis. Our results remain robust to this inclusion such that these controls receive almost no weight in the construction of synthetic Sweden, suggesting that Sweden’s ability to drastically increase healthcare capacity during the pandemic acts as a moderating factor. The overall composition of synthetic Sweden as countries with similarly excellent healthcare systems and thereby also similar pre-pandemic mortality patterns points towards the fact that such pre-existing trends are successfully controlled for and therefor does not explain the observed excess mortality for Sweden.

## Conclusion

Our results on excess mortality cannot recommend that Sweden should have implemented a lockdown as we do not consider the other considerable economic and social costs of a lockdown. A further caveat is that our analysis of Sweden as the treated unit does not directly generalise to government policy responses to pandemics in other countries, as this will depend on various country specific factors such as demographic structure, epidemiological conditions and trust in government, to name a few.

Overall, our results do suggest that not implementing a mandatory lockdown led to higher excess deaths in Sweden during the Covid-19 pandemic. Examining the effect of lockdowns on Covid-19 infections is problematic as detected cases are not independent of the number of tests, while examining Covid-19 specific deaths in this context underestimates the death-toll and at best, provides a partial picture of the effect of not implementing a lockdown. We believe our outcome of examining excess deaths attributed to all causes, provides a more comprehensive estimate of the effect of not implementing a lockdown, by accounting for all deaths caused (eg. avoidance/unavailability of health services, increased CVD deaths) and prevented (eg. automobile accidents, decreased influenza transmission) by disruptions due to the pandemic.

## Materials & methods

Excess mortality is defined as the difference between the observed numbers of deaths in specific time periods and expected numbers of deaths in the same time periods. For excess mortality, we use the Human Mortality Databases’ STMF data series. These provide data on all-cause mortality fluctuations by week, sex and aggregated age group within each calendar year. There are several advantages of using excess deaths over data on infections or covid specific deaths to evaluate the overall impact of covid and the effectiveness of associated lockdown measures. First, data on infections are likely to suffer from measurement error due to missing data on number of tests, imperfection of accuracy of tests and sample selection in that not all covid positive individuals will get tested. Second, data on deaths due to covid is likely to be an underestimate as covid may not always be diagnosed, especially in the earlier months of the pandemic and because of heterogeneous testing policies across countries. Data on excess deaths do not suffer from measurement error arising from these dimensions. Therefore to the extent that the excess mortality has been accurately predicted, these are likely to provide a more accurate picture of the effect of Covid-19 and associated lockdown measures. Our outcomes are 5 year average death counts, and death rates calculated using the Lee-Carter model^28^. Data on control variables were retrieved from the Worldbank (GDP per capita 2020 & Life expectancy 2019) or Our World in Data (Age-standardized death rate cardiovascular 2019, Urbanization 2020 & Population density 2020). To control for life expectancy and age-standardized death rate by cardiovascular disease we utilized 2019 instead of 2020 data as these could be strongly affected by the pandemic itself.

We restrict our analysis to the end of September for four reasons. First, the end of September marked a return of Swedes to workplaces, as compared to synthetic Sweden shown by our analysis of Google mobility reports in Fig. 3. Second, there were significant and considerable policy changes in Sweden by October, 2020, with regards to restrictions. On the 20th of October, Uppsala became the first region to introduce local restrictions and stricter recommendations for workplaces (everyone who can work from home should do so), shops and sports facilities. People were urged to avoid public transport and contact with others who are not members of the same household. While no legal sanctions introduced, these were no longer optional restrictions. Several other regions followed suit, such that within one month, by the 19th of November, 20 out of the 21 regions of Sweden had such local restrictions and recommendations in place. Third, by the 6th of November, the difference between Sweden and Norway, the largest contributor to synthetic Sweden, with regards to their Health and Containment Index (Calculated on the basis of the following thirteen metrics: school closures; workplace closures; cancellation of public events; restrictions on public gatherings; closures of public transport; stay-at-home requirements; public information campaigns; restrictions on internal movements; international travel controls; testing policy; extent of contact tracing; face coverings; and vaccine policy.) was less pronounced^29^. Thus, by November, synthetic Sweden is no longer an appropriate counterfactual for Sweden due to policy changes in the donor countries. We do not attempt a region based SCM analysis as done in other contexts^30,31^ due to low numbers in excess mortality when disaggregated into regions. Finally, yet importantly, it has been shown that estimates for excess mortality, irrespective of the specific method of estimation, struggle to accurately predict mortality patterns especially during influenza season (usually Fall / Winter)^14^, providing yet another reason why we decided to restrict our analysis to the end of September.

### Significance statement

This paper estimates the causal effect of not implementing a lockdown in Sweden on excess deaths. We find that by the end of September, 2020, approximately 10% of all deaths in Sweden could have been avoided, had Sweden implemented a mandatory lockdown. We also find that the effect of not implementing a lockdown on excess mortality was seen in the weeks immediately following, which is in contrast to the existing evidence that examines Covid-19 infections as outcomes. By examining excess deaths attributed to all causes, we provide a comprehensive estimate of the effect of not implementing a mandatory lockdown, by accounting for all deaths caused and prevented by disruptions due to the pandemic.(Supplementary information 1, 2, 3, 4 and 5).

### Supplementary Information

Supplementary Information 1.

Supplementary Information 2.

Supplementary Information 3.

Supplementary Information 4.

Supplementary Information 5.

## Data Availability

The datasets analysed in the current study are publicly available in the Human Mortality Database repository which can be accessed here: https://www.mortality.org/Data/STMF
