# Supplementary figures and images for: The unseen toll: excess mortality during covid-19 lockdowns
Source: Sci Rep. 2023 Oct 31;13:18745. doi: 10.1038/s41598-023-45934-2 (PMC10618514; doi:10.1038/s41598-023-45934-2)

—●— treated —●— estimate —●— placebo

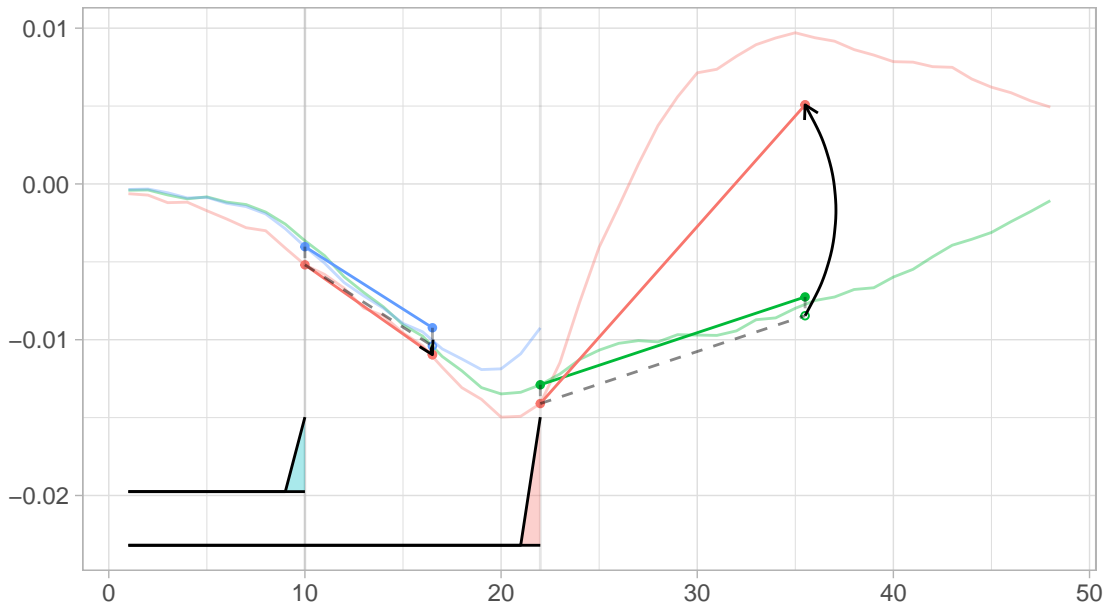

Supplement: Supplementary file 1 — Supplementary Information 1. [file 41598_2023_45934_MOESM1_ESM.zip › sdid_placebor.pdf]

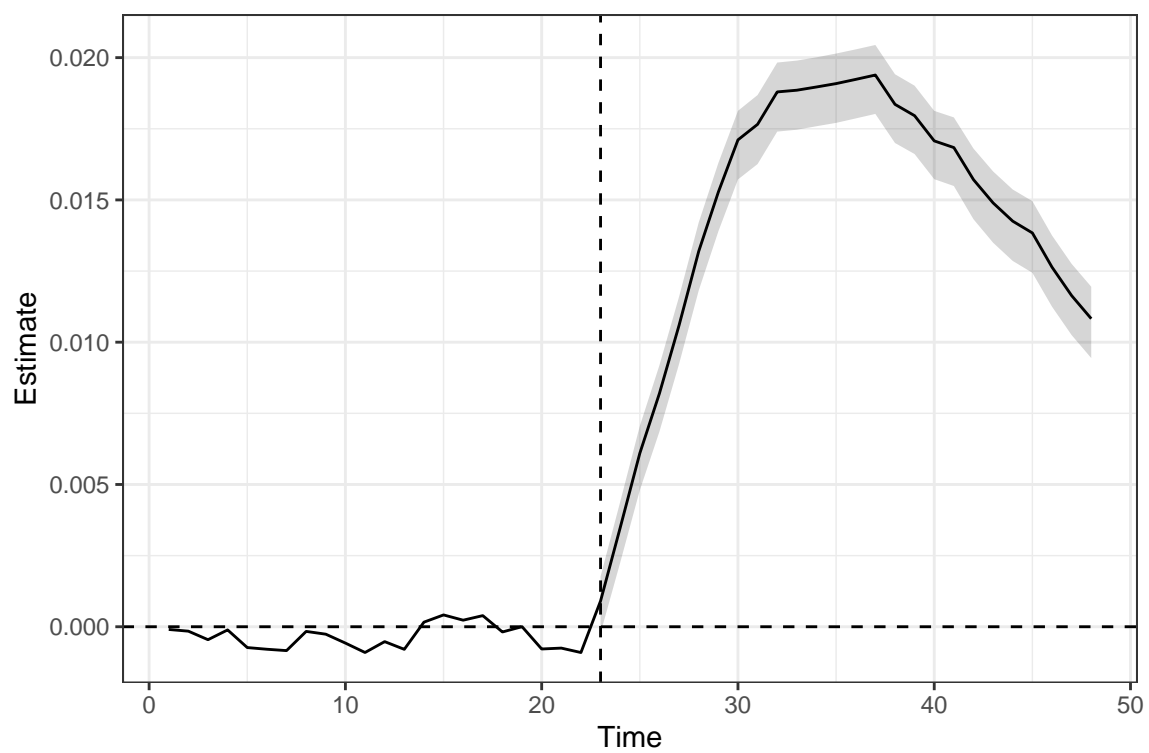

Supplement: Supplementary file 1 — Supplementary Information 1. [file 41598_2023_45934_MOESM1_ESM.zip › augsynth_covr.pdf]

Cumulative Excess Deaths rates

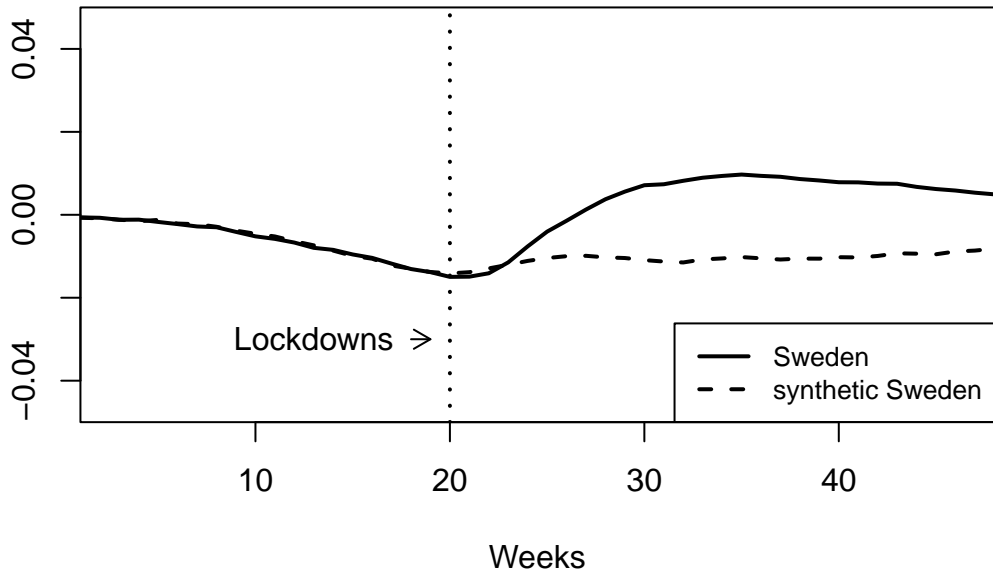

Supplement: Supplementary file 1 — Supplementary Information 1. [file 41598_2023_45934_MOESM1_ESM.zip › CAVG5rPlot20.pdf]

Cumulative Excess Deaths per 100.000

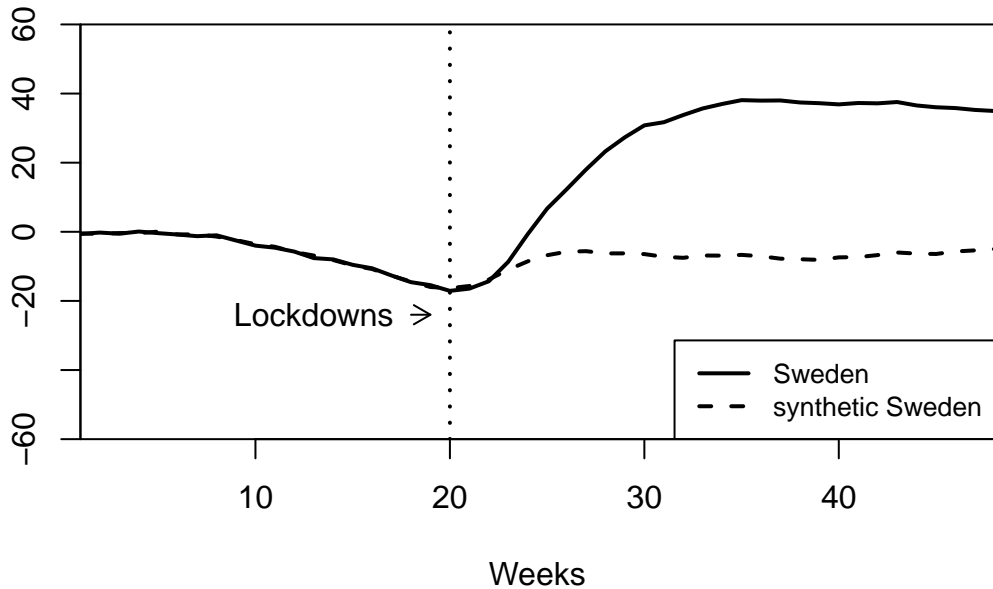

Supplement: Supplementary file 1 — Supplementary Information 1. [file 41598_2023_45934_MOESM1_ESM.zip › CAVG5cPlot20.pdf]

Cumulative Excess Deaths rates

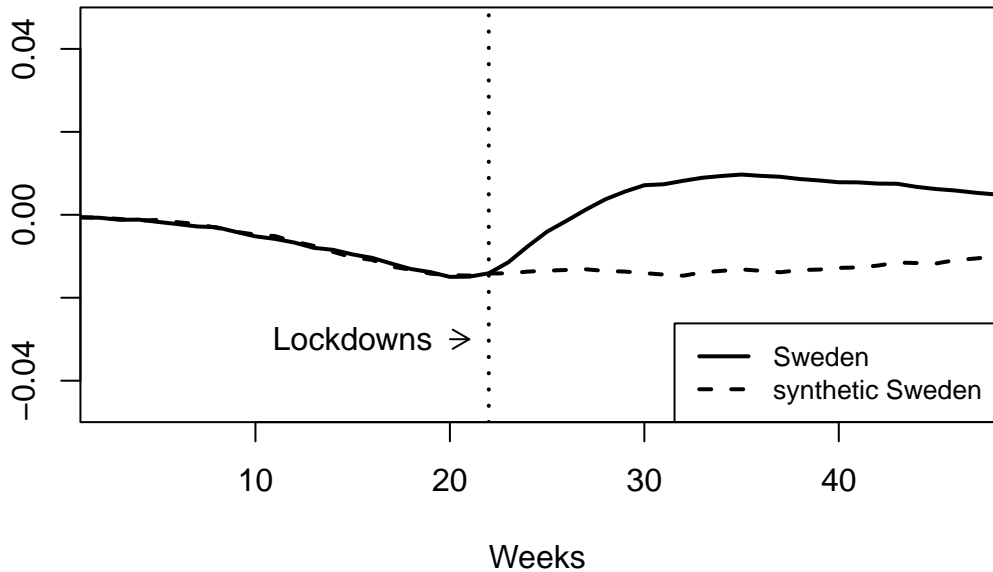

Supplement: Supplementary file 1 — Supplementary Information 1. [file 41598_2023_45934_MOESM1_ESM.zip › CAVG5rPlot22.pdf]

Cumulative Excess Deaths per 100,000

100  
50  
0  
-50  
-100

10

20

30

40

Weeks

Lockdowns >

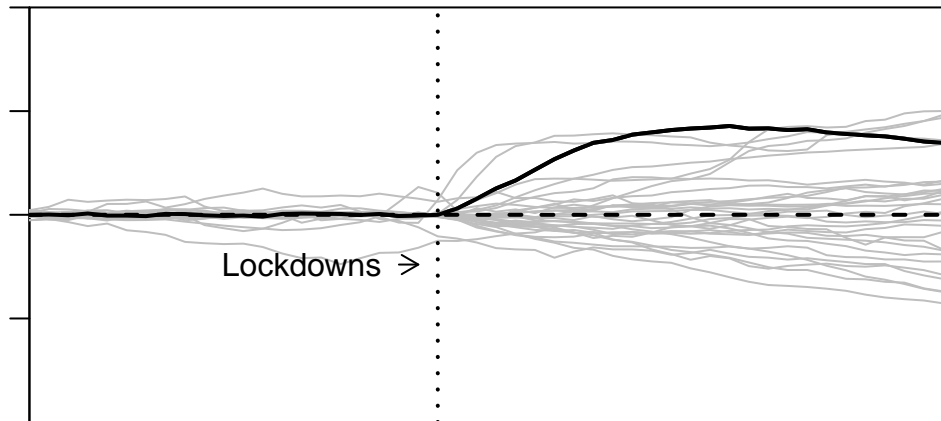

Supplement: Supplementary file 1 — Supplementary Information 1. [file 41598_2023_45934_MOESM1_ESM.zip › CAVG5cPlacebo22.pdf]

Cumulative Excess Deaths per 100,000

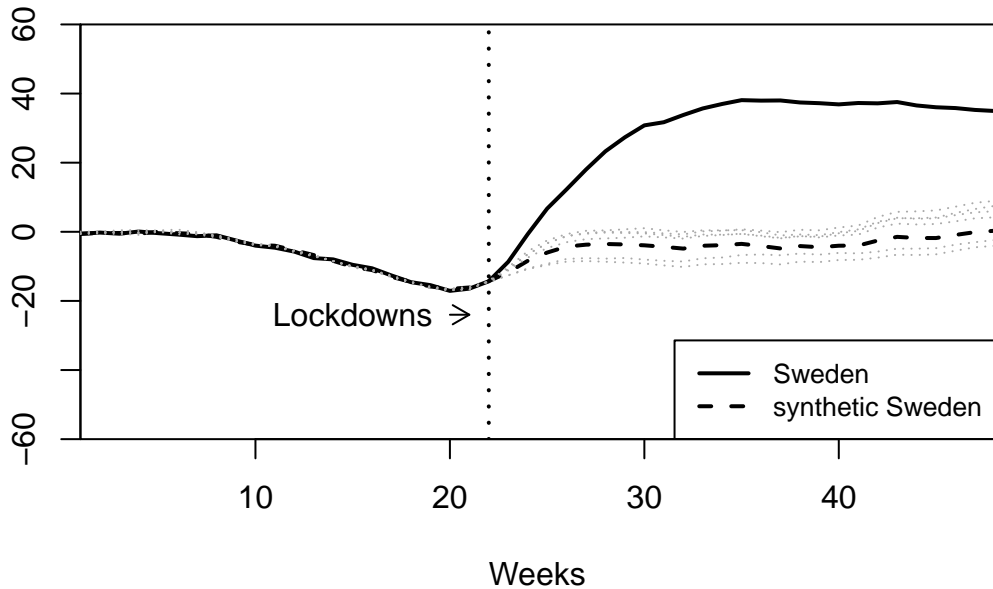

Supplement: Supplementary file 1 — Supplementary Information 1. [file 41598_2023_45934_MOESM1_ESM.zip › CAVG5cLOO22.pdf]

Cumulative Excess Deaths per 100.000

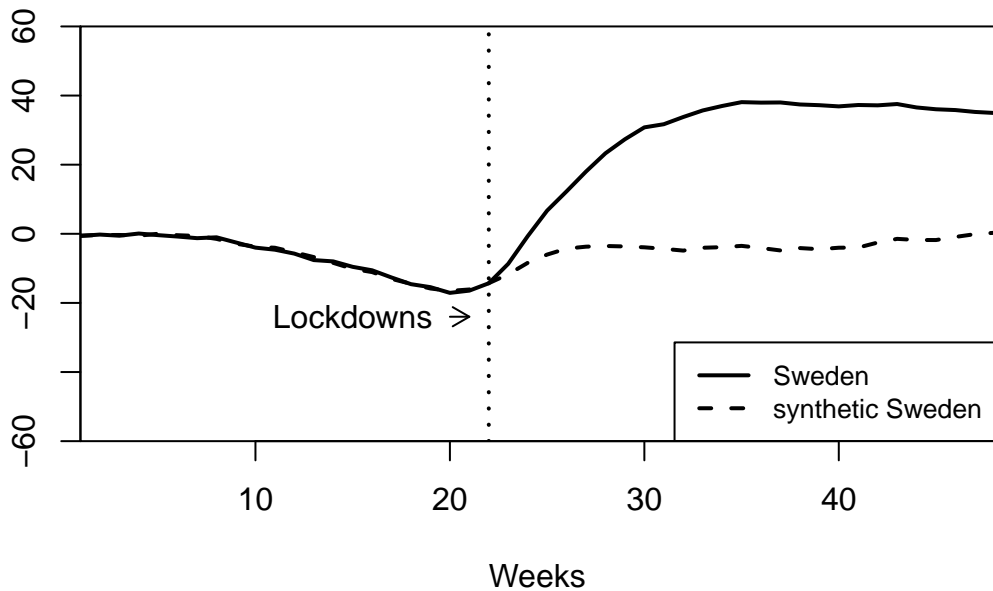

Supplement: Supplementary file 1 — Supplementary Information 1. [file 41598_2023_45934_MOESM1_ESM.zip › CAVG5cPlot22.pdf]

Cumulative Excess Deaths rates

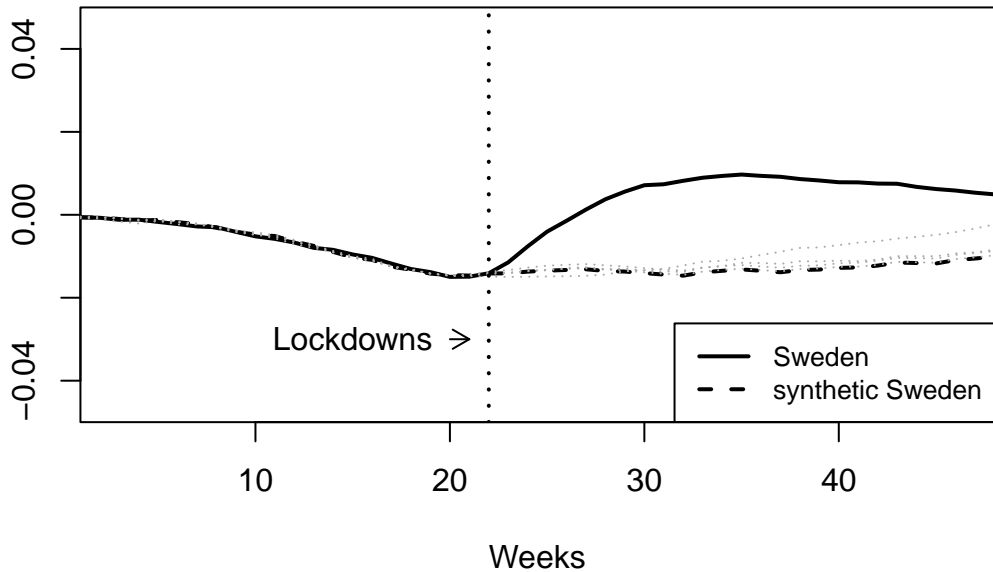

Supplement: Supplementary file 1 — Supplementary Information 1. [file 41598_2023_45934_MOESM1_ESM.zip › CAVG5rLOO22.pdf]

● treated ● estimate ● placebo

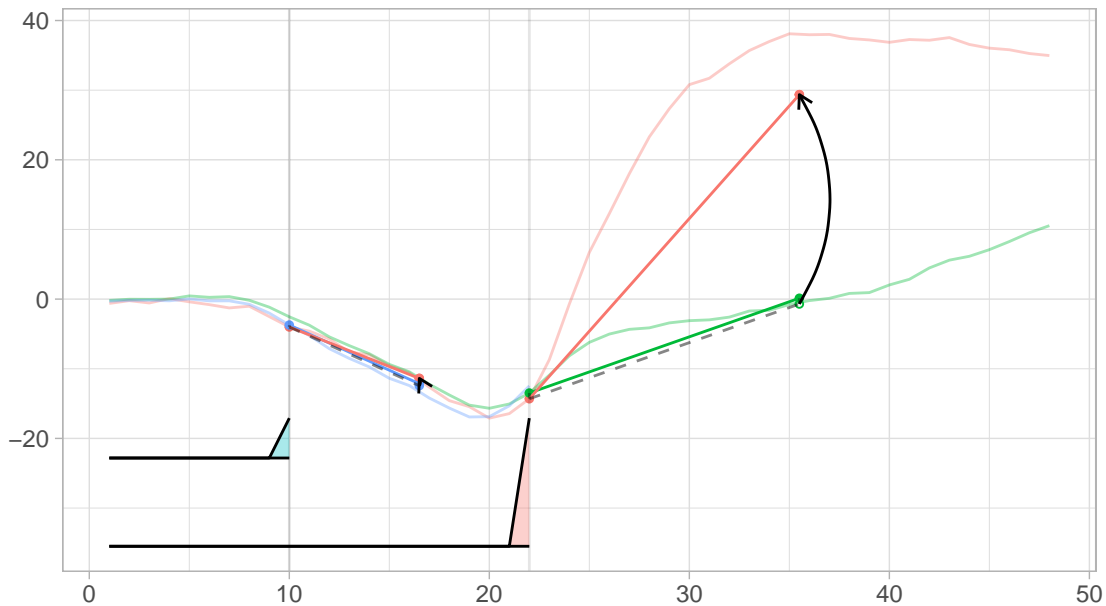

Supplement: Supplementary file 1 — Supplementary Information 1. [file 41598_2023_45934_MOESM1_ESM.zip › sdid_placeboc.pdf]

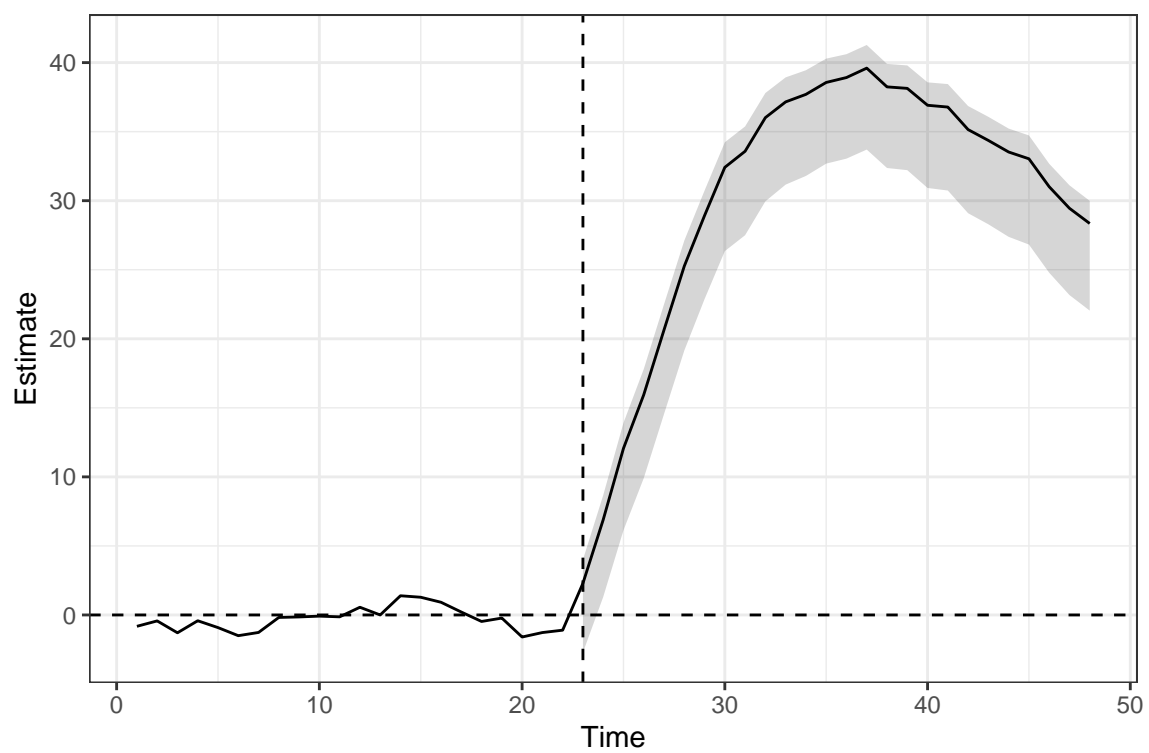

Supplement: Supplementary file 1 — Supplementary Information 1. [file 41598_2023_45934_MOESM1_ESM.zip › augsynth_covc.pdf]

Cumulative Excess Deaths rates

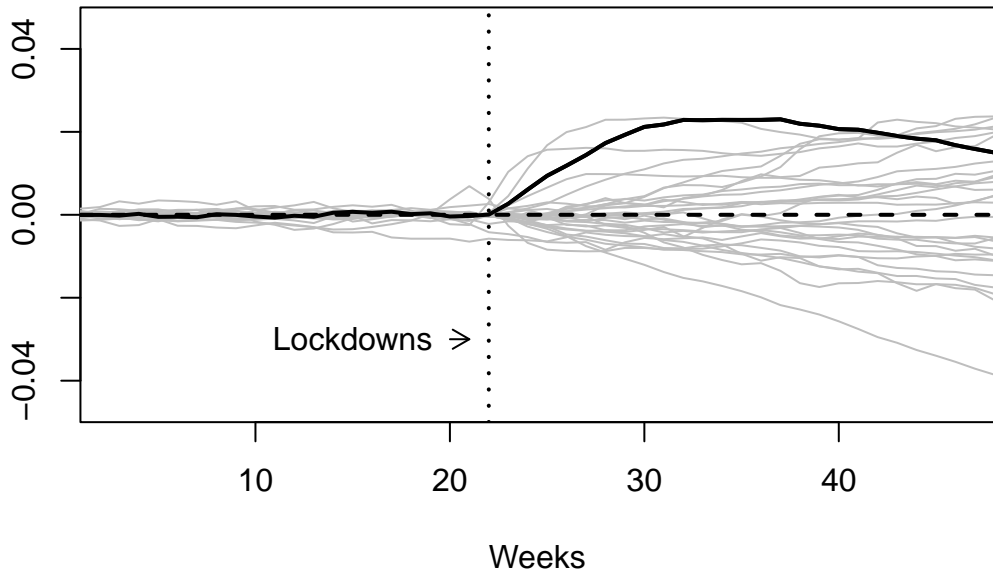

Supplement: Supplementary file 1 — Supplementary Information 1. [file 41598_2023_45934_MOESM1_ESM.zip › CAVG5rPlacebo22.pdf]

Cumulative Excess Deaths per 100,000

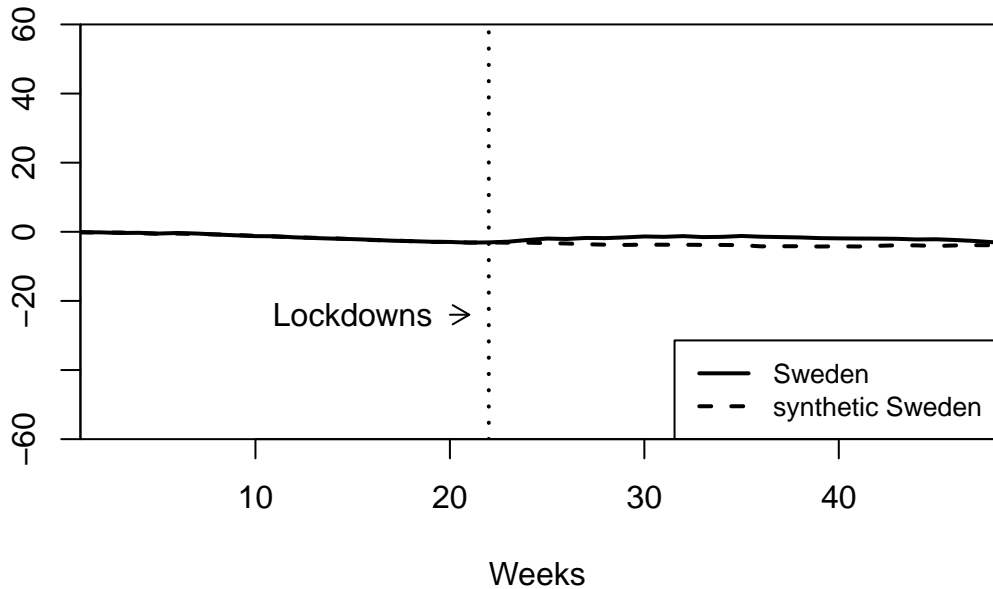

Supplement: Supplementary file 1 — Supplementary Information 1. [file 41598_2023_45934_MOESM1_ESM.zip › CAVG5cPlot0_64.pdf]

Cumulative Excess Deaths per 100,000

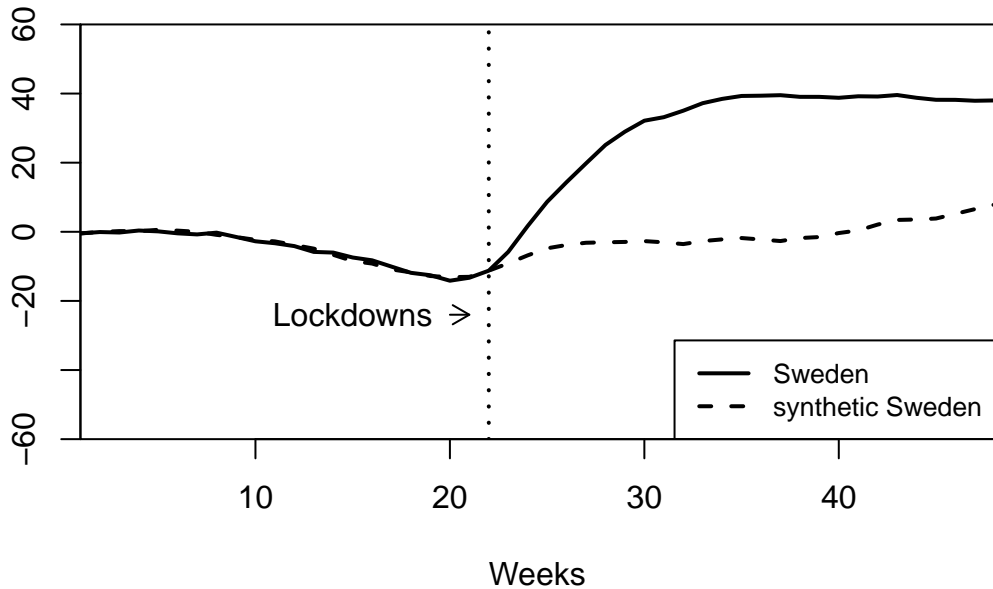

Supplement: Supplementary file 1 — Supplementary Information 1. [file 41598_2023_45934_MOESM1_ESM.zip › CAVG5cPlot65p.pdf]

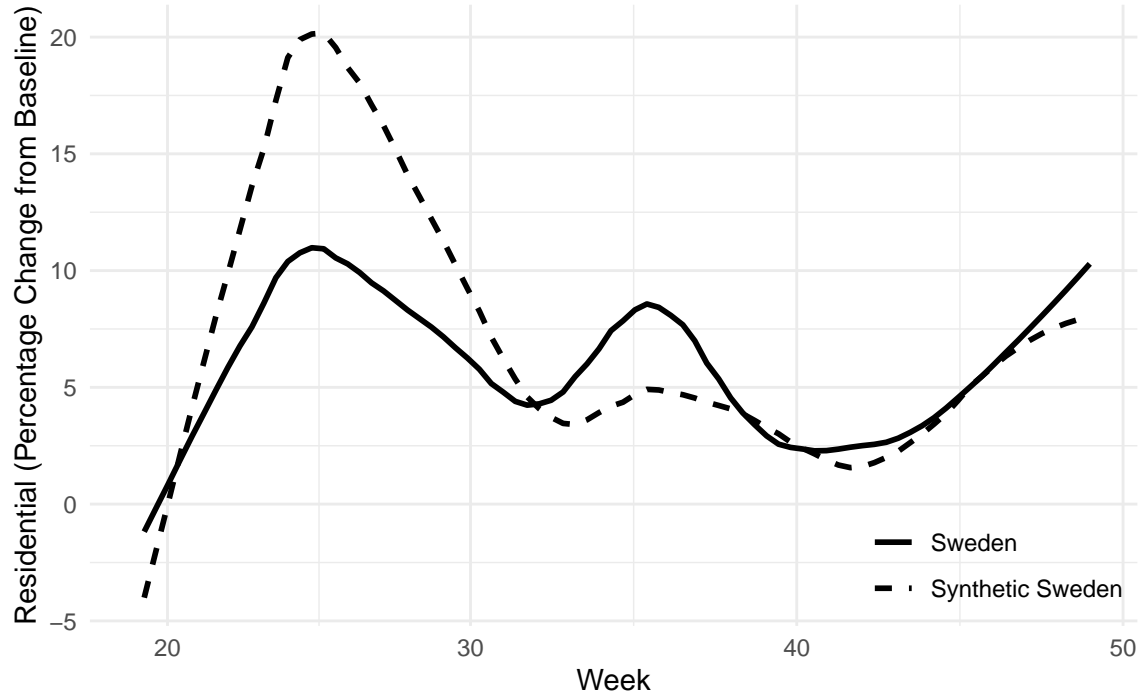

Supplement: Supplementary file 1 — Supplementary Information 1. [file 41598_2023_45934_MOESM1_ESM.zip › MobilityResidential.pdf]

Workplace (Percentage Change from Baseline)

20

30

40

50

Week

— Sweden

- - Synthetic Sweden

0

-20

-40

-60

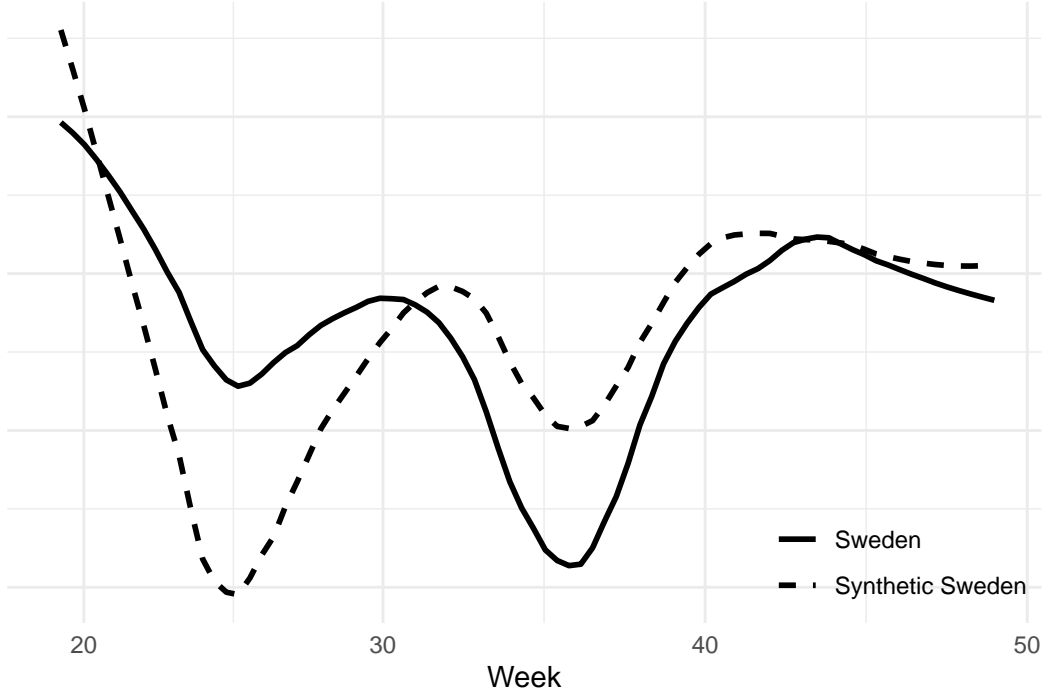

Supplement: Supplementary file 1 — Supplementary Information 1. [file 41598_2023_45934_MOESM1_ESM.zip › MobilityWorkplaces.pdf]
